# Transcriptomic comparison analysis across seven developmental stages of the *Triatoma rubrofasciata*, a vector of Chagas disease

**DOI:** 10.1186/s12864-025-11632-8

**Published:** 2025-05-05

**Authors:** Lei Duan, Yunjia Tian, Ziyi Wang, Limin Yang, Yunhai Guo, Yuanyuan Li, Zhengbin Zhou, Yong Shen, Yi Zhang, Qin Liu

**Affiliations:** 1 NHC Key Laboratory of Parasite and Vector Biology, WHO Collaborating Centre for Tropical Diseases, National Key Laboratory of Intelligent Tracking and Forecasting for Infectious Diseases, National Institute of Parasitic Diseases at Chinese Center for Disease Control and Prevention (Chinese Center for Tropical Diseases Research), National Center for International Research on Tropical Diseases, Shanghai, 200025 People’s Republic of China; 2 State Key Laboratory of Genetic Engineering, Collaborative Innovation Center of Genetics and Development, School of Life Sciences, Fudan University, Shanghai, 200438 People’s Republic of China; 3 Chongqing Jiangjin District Center for Disease Control and Prevention, Chongqing, 402260 People’s Republic of China

**Keywords:** Neglected tropical diseases, Triatomines, *T. rubrofasciata*, Differential gene expression analysis

## Abstract

**Background:**

*Triatoma rubrofasciata* is an obligate hematophagous insect and a primary vector of *Trypanosoma cruzi*, the etiological agent of Chagas disease, with a widespread global distribution. In addition to *Try. cruzi*, *T. rubrofasciata* also serves as a vector for various other pathogens, including *Try. lewisi*, *Try. conorhini*, and *Bartonella* species. Despite its increasing epidemiological relevance in the transmission of multiple diseases, research on *T. rubrofasciata* remains limited.

**Results:**

Differentially expressed genes (DEGs) were associated with growth, development, carbohydrate metabolism, and immunity. Notably, *homeobox* protein genes, including *homeobox protein Nkx-6.2-like*, *homeobox protein abdominal-B isoform X1*, *homeobox protein Hox-A3-like*, and *Hox-B4-like*, along with E3 ubiquitin protein ligase genes and sexual differentiation-related genes, such as *male-specific lethal 1-like 1 isoform X3* (*MSL*), *transformer-2 protein homolog beta-like isoform X2* (*tra-2*), and *doublesex- and mab-3-related transcription factor A2-like* (*dsx*), were highly expressed in the egg stage. Additionally, venom-related genes, including *venom histidine phosphatase-like protein 1* and *venom serine carboxypeptidase-like*, were predominantly expressed in nymphal stages 4 and 5, while *cytochrome P450 CYP425A1v2* exhibited high expression levels in the adult stages. Among these DEGs, we propose that *homeobox* protein genes, *dsx*, *tra-2*, and others may serve as candidate genes involved in growth, development, and sexual differentiation. This study provides valuable insights into gene expression dynamics during *T. rubrofasciata* development and establishes a foundation for future functional research on this species.

**Conclusions:**

In this study, we sequenced the complete developmental stages of *T. rubrofasciata* using HiSeq technology. Our findings offer novel insights into the molecular mechanisms underlying development and sex regulation in this species. Furthermore, the identified differentially expressed genes (DEGs) may serve as potential targets for innovative pest control strategies.

**Supplementary Information:**

The online version contains supplementary material available at 10.1186/s12864-025-11632-8.

## Background

Chagas disease, also known as *American trypanosomiasis*, is a neglected tropical disease caused by the protozoan parasite *Trypanosoma cruzi* [1, 2]. First identified in 1909 by the Brazilian scientist Carlos Ribeiro Justiniano Chagas in Minas Gerais, Brazil, this potentially life-threatening illness has been described by the World Health Organization (WHO) and others as a “silent and silenced disease,” as the majority of infected individuals remain asymptomatic for years before developing severe complications, such as cardiomyopathy and megacolon, in 30–40% of cases [3].

*Try. cruzi* is a flagellated protozoan capable of parasitizing the bloodstream and proliferating within various cell types in humans and other mammals. The parasite is transmitted through multiple routes, including vector-borne transmission, blood transfusion and blood products, mother-to-child vertical transmission, organ transplantation, ingestion of contaminated food and beverages (oral transmission), and laboratory accidents [3]. Despite advancements in disease control, Chagas disease continues to affect an estimated 6–8 million people in the Americas. Furthermore, migration and specific transmission routes have facilitated its spread beyond its traditional geographic range, leading to its recognition as a global health concern in the 21st century [4]. In response to the need for increased awareness and advocacy, the World Health Assembly designated April 14 as World Chagas Disease Day in 2019 [1].

Triatoma (kissing bugs) is a predatory genus of blood-sucking insects belonging to the family Reduviidae, subfamily Triatominae. It is a well-recognized vector in the transmission of *Try. cruzi* [5, 6]. This triatomine undergoes seven developmental stages: eggs, five instar nymphs, and adults [7]. To date, more than 150 *Triatoma* species have been identified as vectors of *Try. cruzi* [5]. Among them, *T. rubrofasciata* is notable for its extensive global distribution and established role in transmitting Chagas disease [5, 6]. This species exhibits high fecundity, requiring a minimum of 82 days to develop from egg to adulthood. It also demonstrates a remarkable capacity for starvation, with fourth-instar nymphs displaying the highest resistance—surviving up to 120 days without food—whereas first-instar nymphs show the lowest resistance, surviving a maximum of 38 days after molting in the absence of a food source [8].

In recent years, the presence of *T. rubrofasciata* has increased significantly across several Asian countries, as evidenced by reports from China [5, 9, 10], Vietnam [8], India [11], and Sri Lanka [12]. A national survey conducted in southern China between 2016 and 2018 confirmed the presence of *T. rubrofasciata* in at least five provinces, where it is commonly found in close association with human habitats [10]. Reports of bites by *T. rubrofasciata* have also risen sharply in several regions of southern China, posing a public health concern due to their potential to trigger severe anaphylactic reactions [9]. Although *T. rubrofasciata* populations in Asia are not infected with *Try. cruzi*, they have been found to carry other trypanosomatid parasites, including *Try. lewisi* and *Try. conorhini* [8, 13], with high infection rates of *Try. conorhini* reported in southern China [13]. *Try. conorhini* and *Try. lewisi* are extracellular kinetoplastid parasites of mammals and are evolutionarily close to *Try. cruzi*. Notably, both species exhibit natural resistance to normal human serum, raising concerns that they may be underestimated pathogens capable of infecting humans [14, 15]. Additionally, *T. rubrofasciata* has been found to harbor bacterial pathogens such as *Bartonella* species in China [16].

It is worth noting that *T. rubrofasciata* possesses 22 autosomes, which is unusual among species of the subfamily *Triatominae*, as other species typically have either 18 or 20 autosomes. The diploid chromosome number of male *T. rubrofasciata* is 25, comprising 22 autosomes and three sex chromosomes (X_1_, X_2_, and Y) [8]. Whether this unique chromosomal structure contributes to its wide adaptability remains unclear. Notably, *T. rubrofasciata* exhibits a high fecundity rate, an exceptional capacity for starvation tolerance, and a high pathogen-carrying potential. Additionally, its close association with human habitats and distinct chromosomal characteristics have garnered increasing attention in China, particularly in the context of vector control strategies. Investigating its growth, development, and sex determination genes will provide further insights into effective *Triatoma* prevention and control.

The transcriptome encompasses all RNA types present in a specific tissue or cell at a given state. With the advent of high-throughput sequencing technologies, transcriptomic analysis has been widely used to identify mRNAs and non-coding RNAs, quantify their expression levels, and explore gene function and structure [17]. Previously, we reported the chromosome-level genome of *T. rubrofasciata* [18]. However, transcriptomic data for this species have remained unavailable until now. In this study, to characterize gene expression patterns across different developmental stages of *T. rubrofasciata*, we sequenced 24 samples representing seven developmental stages using the Illumina sequencing platform. This work aims to elucidate gene expression dynamics throughout *T. rubrofasciata* development and provide valuable transcriptomic resources for future research on gene function, speciation, and phylogenetic relationships in this species.

## Materials and methods

### Insect rearing

*T. rubrofasciata* specimens were collected from the laboratory of the National Institute of Parasitic Diseases, Chinese Center for Disease Control and Prevention. The insects were maintained in an incubator at 28 °C ± 1 °C with a relative humidity of 75% ± 5% and a 12 L:12D photoperiod. Fertilized eggs were collected three days after oviposition. To ensure fertilization, adult males and females were paired under controlled laboratory conditions, and fertilization was confirmed by microscopic examination prior to sample collection. Samples of first- to fifth-instar nymphs, as well as female and male adults, were collected 2–3 days after molting and prior to feeding. Whole bodies were then collected and stored at − 80 °C for RNA extraction. Three biological replicates were obtained for each developmental stage (3 samples for eggs, 3 samples for each of the 5 nymphal instars, and 3 samples each for male and female adult), yielding a total of 24 samples for RNA-seq. Sample identification was confirmed by examining external characteristics as described by Liu et al. [5, 19].

### RNA isolation, cDNA library preparation, illumina sequencing, and transcriptomic analysis

Total RNA was extracted using TRIzol® Reagent (Invitrogen, CA, USA). RNA purity (OD260/280 ≥ 1.8) and integrity (RNA Integrity Number, RIN ≥ 8.0) were assessed using a NanoDrop 2000 spectrophotometer (NanoDrop Technologies, Wilmington, DE, USA) and an Agilent 2100 Bioanalyzer system (Agilent Technologies, CA, USA). mRNA was enriched using Oligo(dT)-attached magnetic beads (NEBNext® Poly(A) mRNA Magnetic Isolation Module, NEB, Cat# E7490) and subsequently fragmented into 200–300 nucleotide fragments via chemical hydrolysis using the NEBNext® Magnesium RNA Fragmentation Module (NEB, Cat# E6150S) at 94 °C for 8 min. First-strand cDNA synthesis was performed with random hexamer primers and SuperScript™ IV Reverse Transcriptase (Invitrogen, Cat# 18090010), followed by second-strand cDNA synthesis using DNA Polymerase I and RNase H as provided in the NEBNext® Ultra™ II RNA Library Prep Kit (NEB, Cat# E7770S). The resulting double-stranded cDNA was purified with Ampure XP beads (Beckman Coulter, CA, USA), and PCR amplification was carried out using NEBNext Ultra II Q5 Master Mix (NEB, Cat# M0544S). Finally, the libraries were quantified with a Qubit 2.0 Fluorometer (Thermo Fisher Scientific) and validated for insert size (250–350 bp) on an Agilent 2100 BioAnalyzer.

Sequencing was performed on an Illumina NovaSeq 6000 platform (Novogene Bioinformatics Institute, Beijing, China) using paired-end 150 bp reads. Raw reads were filtered with SOAPnuke v2.1.0 [20] to remove adapters, low-quality reads (Qphred ≤ 20 in > 50% of bases), and reads with ambiguous bases (N content > 0.5%). Clean reads were mapped to the reference genome (PRJNA516044) [18] utilizing HISAT2 (v2.1.0) [21] with default parameters. RSEM (v1.3.1) [22] was employed for quantification to obtain TPM (Transcripts Per Million) [23]. Principal component analysis (PCA) of TPM for all samples was performed using the prcomp function in R [24].

### RT-qPCR analysis

Total RNA was extracted from different developmental stages of *T. rubrofasciata*, including egg, third-instar nymph, fifth-instar nymph, and adult males and females. Samples were homogenized in liquid nitrogen, and RNA extraction was performed using the RNeasy Plus Mini Kit (Qiagen, Valencia, CA, USA). To eliminate potential genomic DNA contamination, RNA was treated with DNase I (Qiagen). The purity and concentration of RNA were assessed using a NanoDrop 2000 spectrophotometer (NanoDrop Technologies, DE, USA), while RNA integrity was evaluated using an Agilent 2100 Bioanalyzer (Agilent Technologies, CA, USA). Complementary DNA (cDNA) synthesis was carried out with the PrimeScript™ RT Reagent Kit with gDNA Eraser (Takara Bio, Japan).

mRNA quantification was performed using a Bio-Rad C1000 real-time PCR system under the following thermal cycling conditions: an initial denaturation at 95 °C for 3 min, followed by 40 cycles of denaturation at 95 °C for 10 s, annealing at 60 °C for 30 s, and extension at 65 °C for 5 s, with a final extension at 95 °C for 50 s. Each 20 μL reaction mixture contained 10 μL Green qPCR SuperMix, 0.5 μL of 10 mmol/L forward and reverse primers, 1 μL of 10× diluted cDNA, and 8 μL of nuclease-free water. Three biological replicates were analyzed for each developmental stage, with each sample and negative control assessed in triplicate. Primer sequences are provided in Supplementary Table 1.

Gene expression levels were normalized to glyceraldehyde-3-phosphate dehydrogenase (*GAPDH*) as an internal reference. Relative expression was determined using the delta Ct (ΔCt) method [25], based on mRNA quantification from all samples, each with three technical replicates. To evaluate gene expression dynamics across different developmental stages of *T. rubrofasciata*, Ct values were analyzed using the 2^−ΔΔCt^ method. Statistical significance was assessed using the Least Significant Difference (LSD) multiple comparison test, and the results were visualized in a column chart.

### Differential gene expression, gene ontology and KEGG analysis

Differential expression analysis was performed using DESeq2 (v3.11) to identify differentially expressed genes (DEGs) through pairwise comparisons across all developmental stages [26]. Genes were considered significantly differentially expressed if they exhibited an absolute log2 fold change (logFC) ≥ 1 with a false discovery rate (FDR) ≤ 0.05, adjusted via the Benjamini-Hochberg (BH) method [27].

Homology searches against KEGG database were executed using DIAMOND blastp (v2.0.14) [28], enabling precise KEGG Orthology (KO) assignment through reciprocal best-hit methodology. KOBAS 3.0 (-t blastout: tab -s ko) [29] subsequently mapped these KO identifiers to biological pathways while implementing Benjamini-Hochberg FDR correction to ensure statistical validity of pathway associations. Complementary functional characterization was achieved via InterProScan 5 (v5.61-93.0) [30], which systematically interrogated protein sequences against InterPro member databases (Pfam, SMART, PANTHER, etc.) using optimized parameters (--seqtype p --goterms --pathways). This analysis generated Gene Ontology (GO) annotations through domain-specific evidence codes while implementing automated quality controls including taxonomic filtering and removal of obsolete GO terms. Functional enrichment analysis was conducted using the clusterProfiler package (v4.14.6) [31].

### Gene co-expression network analysis

Weighted gene co-expression network analysis (WGCNA) was conducted using the WGCNA R package to explore co-expression patterns. The mad function in R was applied to preprocess the gene expression matrix, retaining the top 75% of genes based on median absolute deviation (MAD), with a minimum threshold of 0.01. Following this filtering step, 9,512 genes were included in the subsequent analysis. The optimal soft-thresholding power was determined automatically. Hub genes were identified based on a module membership (MM) threshold of > 0.9. The resulting co-expression network was visualized using Cytoscape (v3.7.2) (https://cytoscape.org/) [32].

## Results

### Statistics of sequencing data

**Fig. 1:**
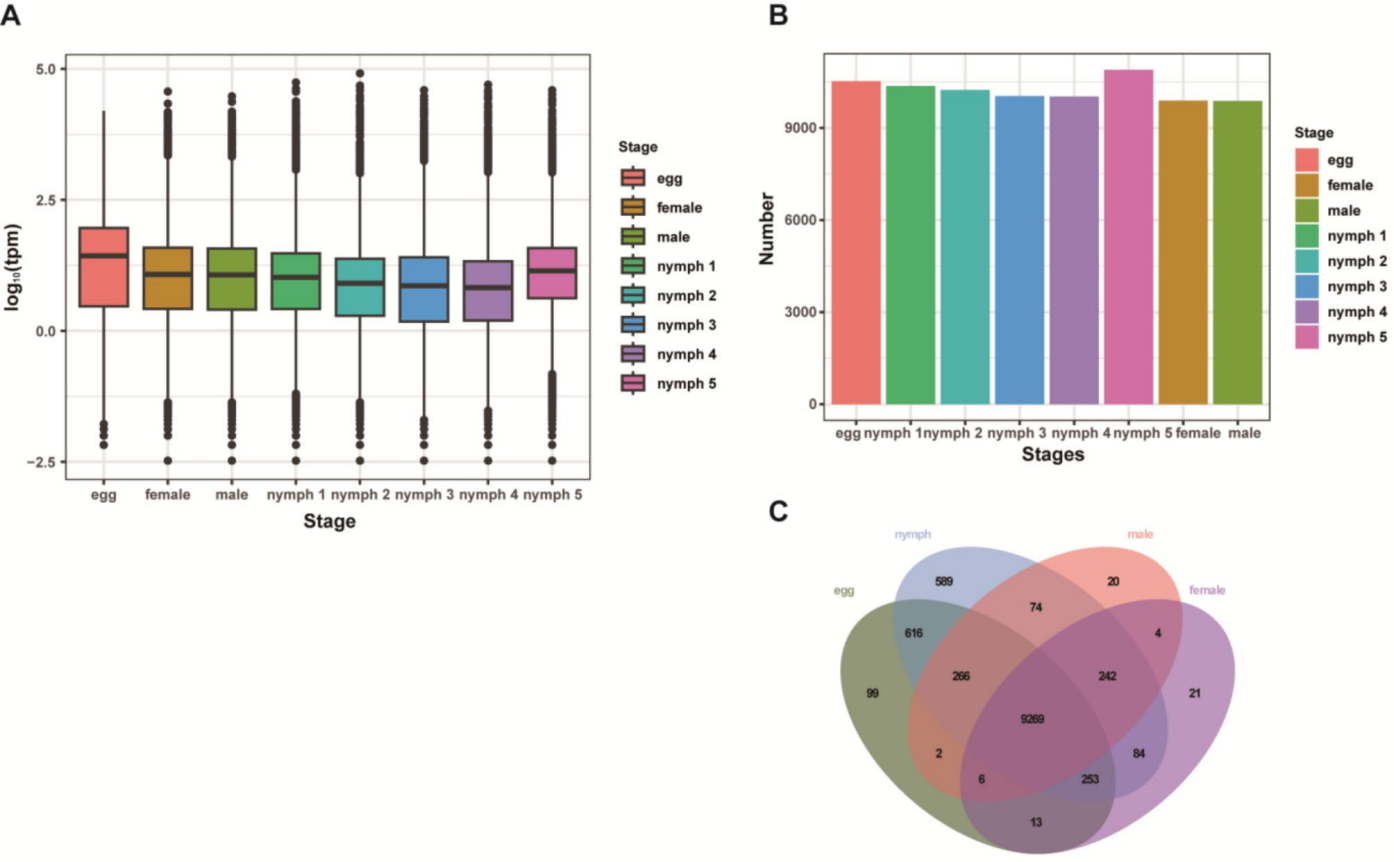
(**A**) Boxplot depicting gene expression levels across different developmental stages of *T. rubrofasciata*. X-axis: Sample groups; Y-axis: Log10 (TPM). (**B**) Gene expression levels across developmental stages. X-axis: Sample groups; Y-axis: Number of expressed genes. (**C**) Venn diagram illustrating the number of genes specifically expressed in different tissues and developmental stages

A total of 170.2 Gb of clean bases and 1,140.9 million clean reads were obtained, with an average total mapping ratio of 89.7% (Supplementary Data 1). Given the *T. rubrofasciata* reference genome size (680.73 Mb) [18], the sequencing depth reached approximately 10.42× per sample, meeting the standard requirements for transcriptome-wide analyses in insects. The high mapping efficiency, along with the validation of differentially expressed genes (DEGs) via RT-qPCR (Fig. 6), confirms the adequacy of sequencing coverage for downstream analyses. Principal component analysis (PCA) effectively distinguished egg stages, nymphal stages, and adult males and females into distinct clusters (Supplementary Fig. 1). Gene expression comparisons revealed that adult males and females exhibited fewer total expressed genes, whereas the egg stage displayed a higher average expression level but a lower number of genes with very high expression (Fig. 1A, B; Supplementary Data 2, 11). Additionally, 99 genes were uniquely expressed in the egg stage, while 589 were specific to all nymphal stages. Adult males and females had 20 and 21 uniquely expressed genes, respectively (Fig. 1C).

### Differentially expressed genes analysis

**Fig. 2:**
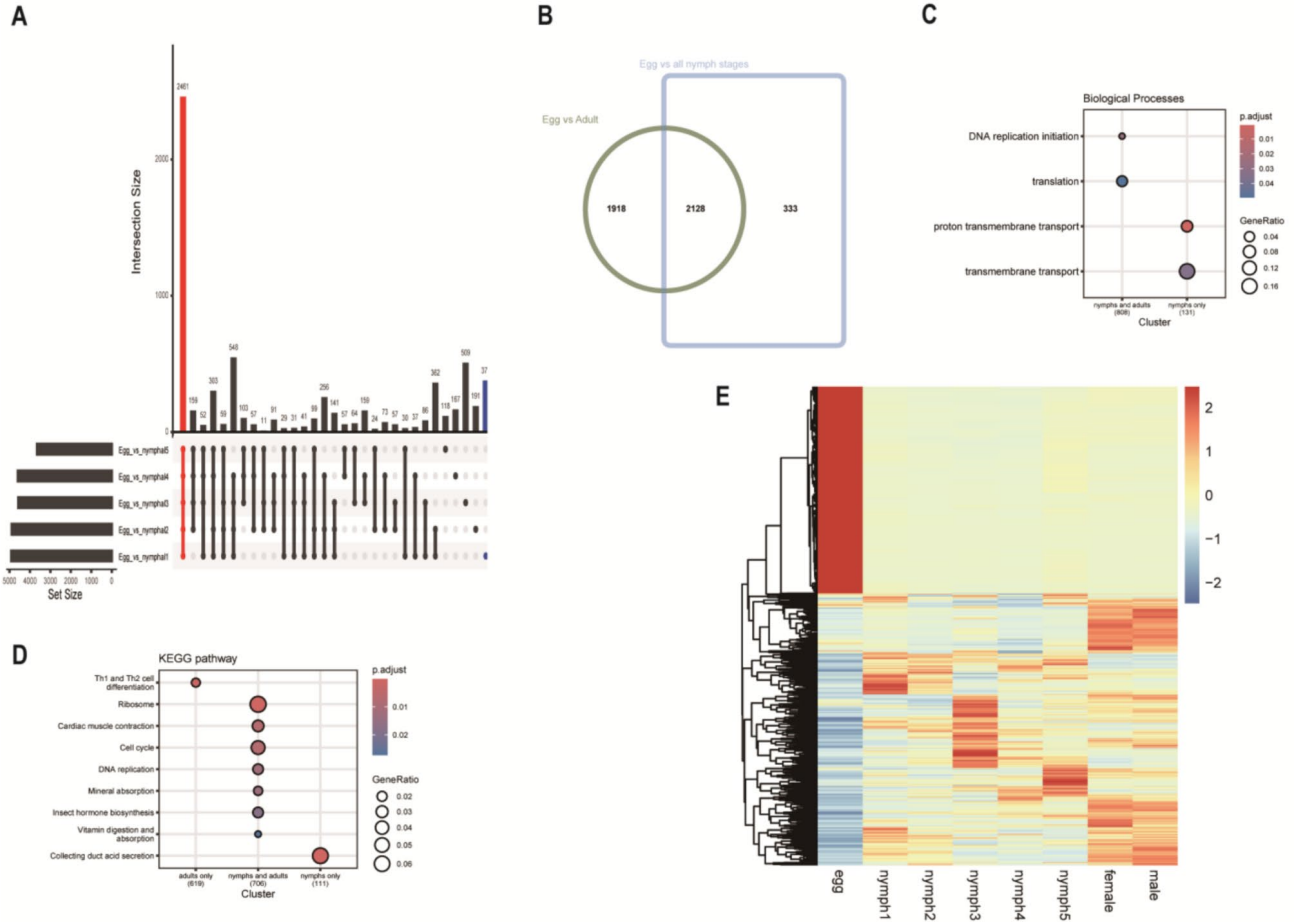
(**A**) Upset plot of differentially expressed genes (DEGs). Set size: Total number of DEGs; Intersection size: Number of overlapping DEGs. (**B**) Overlap of DEGs across egg, nymphal, and adult developmental stages. (**C**) Gene Ontology (GO) enrichment analysis of overlapping gene sets and a pairwise comparison between egg and nymph stages. (**D**) Differential KEGG pathway enrichment between adult and nymphal stages. (**E**) Heatmap illustrating DEGs between the egg stage and other developmental stages

The analysis of differentially expressed genes (DEGs) between the egg stage and all other developmental stages revealed the highest number of DEGs in the early nymphal stages, with 4,913 and 4,892 DEGs identified in comparisons between eggs and nymph stages 1 and 2, respectively (Fig. 2A; Table 1, Supplementary Data 3). This finding suggests substantial transcriptional reprogramming during the early transition from egg to nymph. The number of DEGs progressively declined in later nymphal stages. Comparisons between eggs and adult stages identified 4,527 and 4,588 DEGs in females and males, respectively, indicating significant but relatively similar transcriptional changes associated with adult differentiation.

**Table 1:** Pairwise comparison of the number of differentially expressed genes across developmental stages and between sexes in *T. rubrofasciata* samples

Compared group	Up-regulated gene number	Down-regulated gene number	Total different gene number
egg-vs-nymph1	2734	2179	4913
egg-vs-nymph2	2675	2217	4892
egg-vs-nymph3	2420	2152	4572
egg-vs-nymph4	2510	2083	4593
egg-vs-nymph5	2099	1558	3657
egg-vs-male	2395	2132	4527
egg-vs-female	2382	2206	4588
nymph1-vs-nymph2	329	255	584
nymph1-vs-nymph3	1168	1280	2448
nymph1-vs-nymph4	878	729	1607
nymph1-vs-nymph5	398	650	1048
nymph2-vs-nymph3	821	1025	1846
nymph2-vs-nymph4	335	254	589
nymph2-vs-nymph5	286	521	807
nymph3-vs-nymph4	479	297	776
nymph3-vs-nymph5	474	422	896
nymph4-vs-nymph5	9	29	38
female vs. nymph1	1327	1054	2381
female vs. nymph2	1079	963	2042
female vs. nymph3	693	515	1208
female vs. nymph4	743	690	1433
female vs. nymph5	821	836	1657
female_vs_male	5	8	13
male vs. nymph1	1464	1064	2528
male vs. nymph2	1244	932	2176
male vs. nymph3	750	619	1369
male vs. nymph4	923	772	1695
male vs. nymph5	942	842	1784

A total of 4,046 DEGs were detected in the comparison between eggs and adults, whereas 2,461 DEGs were identified in the comparison between eggs and all nymphal stages, with 2,128 DEGs shared between the nymphal and adult stages (Fig. 2B, Supplementary Data 4). Gene Ontology (GO) enrichment analysis revealed that DNA replication and translation were significantly enriched in the overlapping set, whereas transmembrane transport-related pathways were specifically enriched in the egg vs. nymph comparison (Fig. 2C, Supplementary Data 4). Kyoto Encyclopedia of Genes and Genomes (KEGG) pathway analysis suggested that adult stages prioritize immune regulation, whereas nymphal stages focus on growth and metabolic balance. Shared pathways, particularly those related to hormone biosynthesis, underscore the continuity of developmental processes across stages, despite variations in expression levels (Fig. 2D, Supplementary Data 4).

A heatmap analysis of DEGs present across all comparisons between the egg stage and other developmental stages identified 918 DEGs that were upregulated specifically during the egg stage. These genes were enriched in additional biological processes, such as phosphorylation (Fig. 2E, Supplementary Fig. 2A, Supplementary Data 4). KEGG analysis further suggested that these DEGs are associated with fundamental cellular processes related to growth, maintenance, and responses to stress or damage (Supplementary Fig. 2B, Supplementary Data 4). Pathways such as cell cycle regulation, DNA replication, and ribosome biogenesis indicated active cellular proliferation and protein synthesis, essential for development and tissue renewal. Additionally, the enrichment of glycosaminoglycan biosynthesis pathways suggests a potential role in extracellular matrix formation and signaling, which may contribute to developmental and structural transitions.

**Fig. 3:**
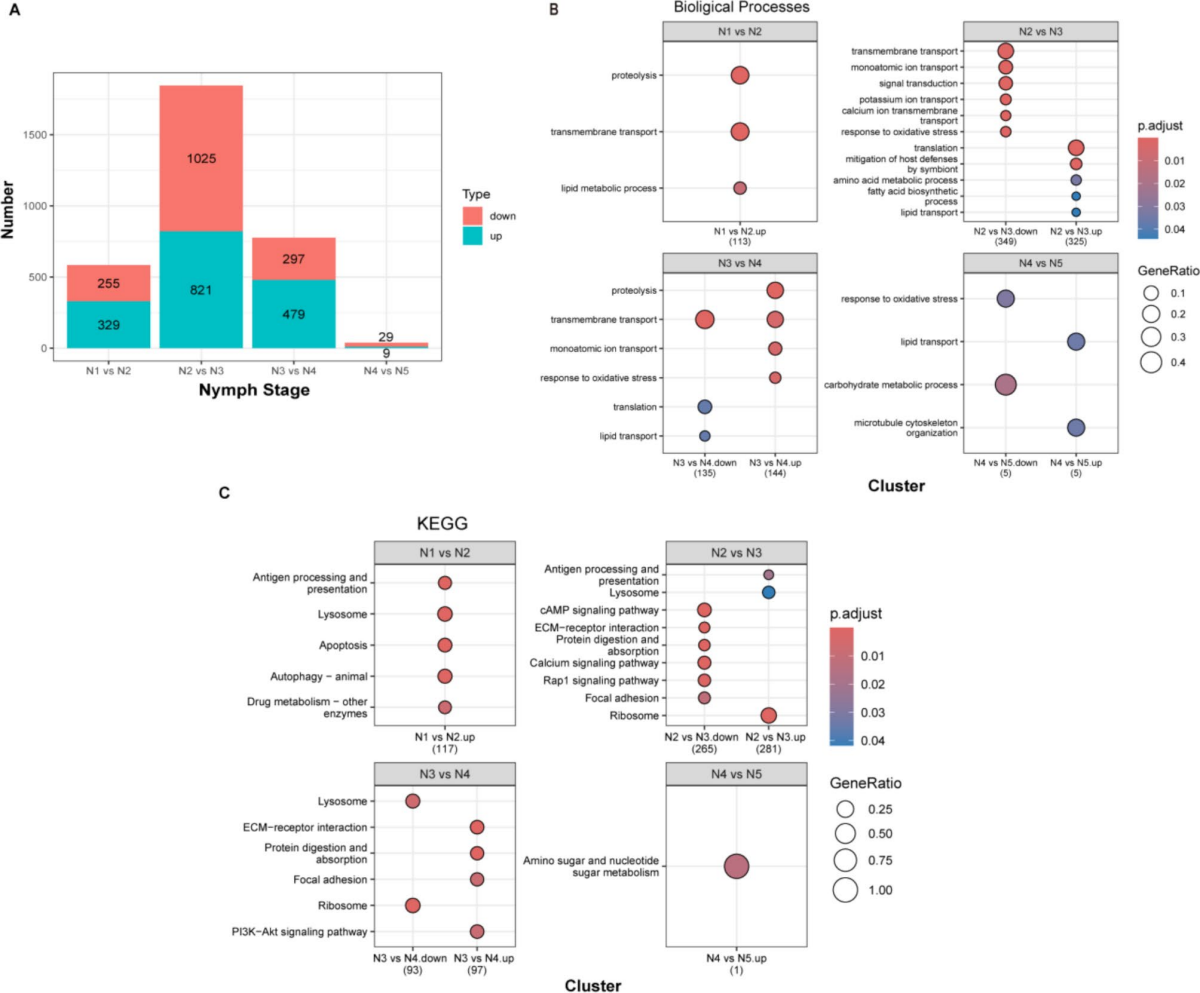
(**A**) Dynamic transcriptional changes across nymphal developmental stages. (**B**) GO enrichment analysis of stage-specific DEGs. (**C**) KEGG enrichment analysis of stage-specific DEGs. GO pathway analysis, X-axis: Different paired groups, Y-axis: GO terms. KEGG pathway analysis, X-axis: Different paired groups, Y-axis: KEGG terms; The p.adjust is represented by the color scale, and the gene ratio by point size. Developmental stages are labeled as follows: E (egg), N1 (nymph 1), N2 (nymph 2), N3 (nymph 3), N4 (nymph 4), N5 (nymph 5), F (female adult), and M (male adult)

A stage-wise comparison of differentially expressed genes (DEGs) across nymphal development revealed dynamic transcriptional changes (Fig. 3A, Supplementary Data 3). The transition from stage 2 to stage 3 exhibited the most pronounced shift, with 1,025 genes downregulated and 821 upregulated, suggesting a major transcriptional reprogramming event. In contrast, the earlier transition (stage 1 to stage 2) involved fewer DEGs (255 downregulated, 329 upregulated), indicating a more gradual change at this stage. The number of DEGs continued to decline in later transitions, with minimal transcriptional differences observed between stage 4 and stage 5 (29 downregulated, 9 upregulated). Gene Ontology (GO) analysis identified consistent enrichment of various transport-related pathways, including monoatomic ion and lipid transport, across most comparisons. Additionally, the response to oxidative stress pathway was enriched in all comparisons except for stage 1 vs. stage 2, suggesting potential stage-specific regulatory mechanisms (Fig. 3B, Supplementary Data 5). KEGG pathway enrichment analysis further highlighted dynamic shifts in metabolic, transport, and stress response processes throughout nymphal development (Fig. 3C, Supplementary Data 5). Early transitions (nymph 1 to nymph 2) were characterized by enrichment in protein degradation and lipid metabolism pathways. Mid-stage transitions (nymph 2 to nymph 3) showed a complex shift, with a reduction in transport and signaling pathways but an increase in protein synthesis and metabolism. Later developmental stages (nymph 3 to nymph 4) exhibited a balance between protein breakdown, ion transport, and stress responses, while the final transition (nymph 4 to nymph 5) was marked by a shift towards lipid transport and cytoskeletal organization, with a reduced emphasis on carbohydrate metabolism.

**Fig. 4:**
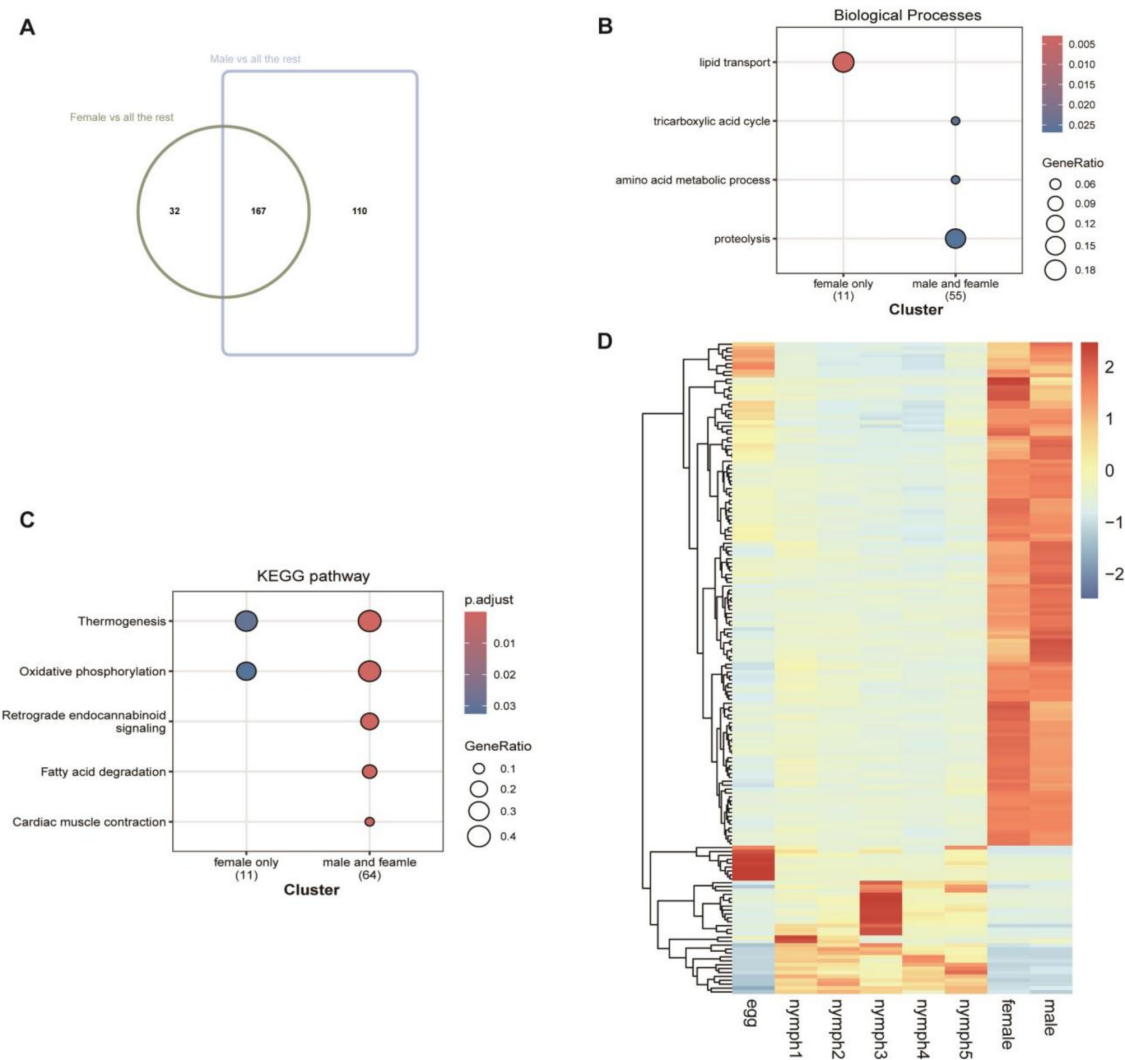
(**A**) Overlap of DEGs between female and male adults compared to all other developmental stages. (**B**) GO enrichment analysis of sex-specific and shared biological processes. (**C**) KEGG enrichment analysis of sex-specific and shared biological processes. (**D**) Heatmap illustrating DEGs between adult and other developmental stages

In adults, fewer differentially expressed genes (DEGs) were identified when comparing adults to all other developmental stages, relative to other pairwise comparisons. Specifically, 199 DEGs were detected in female adults compared to all other stages, while 277 DEGs were identified in male adults. Notably, male adults exhibited a greater number of DEGs than females, with 167 genes overlapping between the two groups (Fig. 4A, Supplementary Data 6). Gene Ontology (GO) analysis revealed both sex-specific and shared biological processes, with lipid transport identified as a female-specific function, whereas energy and protein metabolism were common across both sexes (Fig. 4B, Supplementary Data 6). Kyoto Encyclopedia of Genes and Genomes (KEGG) pathway analysis highlighted a core dependence on energy metabolism in both sexes, with females potentially exhibiting an enhanced thermogenic and oxidative capacity. The shared pathways suggest common requirements for energy production, immune function, and metabolic regulation, likely supporting general survival and adaptation, while female-specific pathways may be associated with unique physiological roles (Fig. 4C, Supplementary Data 6). Among the 167 overlapping DEGs, 129 exhibited significantly higher expression in adults and were enriched in the tricarboxylic acid (TCA) cycle in GO enrichment analysis, whereas KEGG pathway analysis yielded results consistent with the total gene set (Fig. 4D, Supplementary Fig. 3A, 3B, Supplementary Data 6).

### WGCNA analysis

**Fig. 5:**
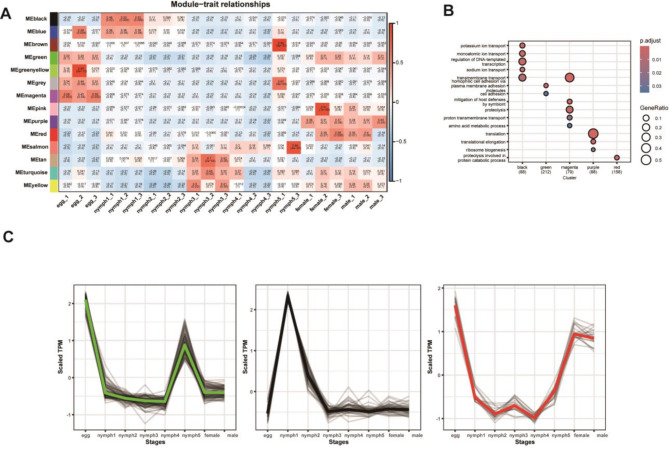
(**A**) Correlation between gene modules and developmental stages of *T. rubrofasciata*. (**B**) GO enrichment analysis of gene modules (X-axis: Module color groups; Y-axis: GO terms). (**C**) Expression patterns of hub genes in the green, black, and red modules across developmental stages (X-axis: Tissues and developmental stages; Y-axis: Scaled TPM)

WGCNA was performed on all RNA-seq data using a soft-thresholding power of 16 (Supplementary Fig. 4). A total of 14 gene co-expression modules were identified from 9,512 filtered expressed genes (Supplementary Fig. 5, Supplementary Data 7). Among these, several modules exhibited strong correlations with specific developmental stages (Fig. 5A). Notably, the magenta and green modules were more closely associated with the egg stage than other modules, whereas the black module showed a stronger correlation with the first nymphal stage compared to the blue module. In adults, including both males and females, gene expression was predominantly linked to the purple and red modules. GO enrichment analysis revealed that genes in the magenta module were primarily involved in transport-related pathways, while those in the green module were enriched in adhesion-related pathways. Similarly, the black module was enriched in ion transport-related pathways, akin to the magenta module. In contrast, the purple module was specifically associated with translation-related processes, including translational elongation and ribosome biogenesis (Fig. 5B, Supplementary Data 8).

### Hub genes and co-expression network

Hub genes, defined as nodes with the highest intramodular connectivity (kME) within co-expression networks, play crucial roles in regulating module functionality due to their central topological positions. Genes with connectivity values greater than 0.9 were identified (Supplementary Data 9). Figure 5C presents the expression patterns of the green, black, and red modules, which exhibit strong associations with specific developmental stages. Hub genes in the green module are predominantly expressed during the egg stage, those in the black module are specifically upregulated in the first nymph stage, and hub genes in the red module show increased expression in both the egg and adult stages.

To further elucidate the co-expression networks of these hub genes, genes with weights greater than 0.95 in relation to the hub genes were selected. In the green module, 93 hub genes and 183 associated genes were identified, with protein phosphorylation emerging as the most prevalent Gene Ontology (GO) term, despite not being highlighted in previous GO enrichment analyses (Supplementary Fig. 6A). In the black module, 47 hub genes and 137 related genes were mapped, with transmembrane transport identified as the most frequently occurring GO term, consistent with the enrichment analysis of the entire module (Supplementary Fig. 6B). For the red module, 20 hub genes and 164 related genes were identified, including eight genes involved in proteolysis, which share GO terms with previous enrichment results (Supplementary Fig. 6C, Supplementary Data 10).

### RT-qPCR validation of different developmental stages

**Fig. 6:**
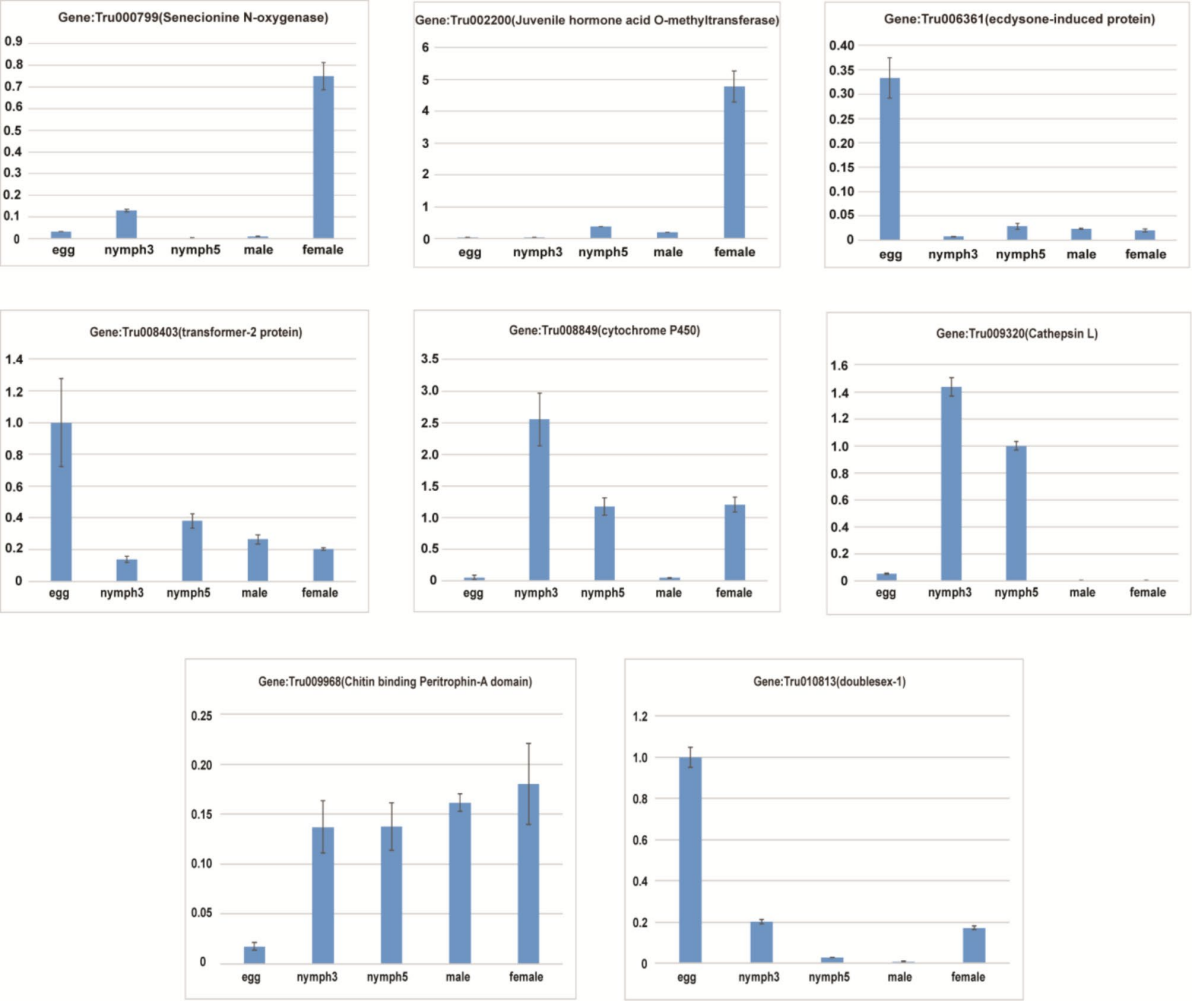
RT-qPCR validation of eight differentially expressed genes identified in the transcriptome across developmental stages

To assess the reliability of the transcriptome sequencing data, GAPDH was used as an internal reference gene. Eight differentially expressed genes (DEGs) identified through RNA-seq were randomly selected for validation via quantitative real-time PCR (RT-qPCR). As shown in Fig. 6, the RT-qPCR results largely corroborated the RNA-seq findings, exhibiting similar expression patterns for most selected genes. Notably, chitinase-3 followed the same trend in both RT-qPCR and RNA-seq but did not show significant changes in the third and fifth nymphal stages when compared to RNA-seq data (Table 2).

**Table 2:** TPM values of eight randomly selected genes validated by RT-qPCR across different developmental stages

Gene ID	egg	nymph3	nymph5	male	female
Gene: Tru000799(Senecionine N-oxygenase)	0.12	63.59	0.22	23.26	532.46
Gene: Tru002200(Juvenile hormone acid O-methyltransferase	0.00	6.75	14.32	14.94	57.42
Gene: Tru006361(ecdysone-induced protein)	2024.12	55.33	90.49	73.25	65.52
Gene: Tru008403(transformer-2 protein)	639.90	55.42	137.26	71.34	79.43
Gene: Tru008849(cytochrome P450)	28.35	12687.81	6391.62	375.64	4211.66
Gene: Tru009320(Cathepsin L)	42.40	42338.97	5135.24	2.37	2.35
Gene: Tru009968(Chitin binding Peritrophin-A domain)	1.12	9.11	15.96	32.78	41.40
Gene: Tru010813(doublesex-1)	4.74	0.09	0.72	0.74	1.12

### Stage-specific expression dynamics of development-related genes across ontogenetic transitions

**Fig. 7:**
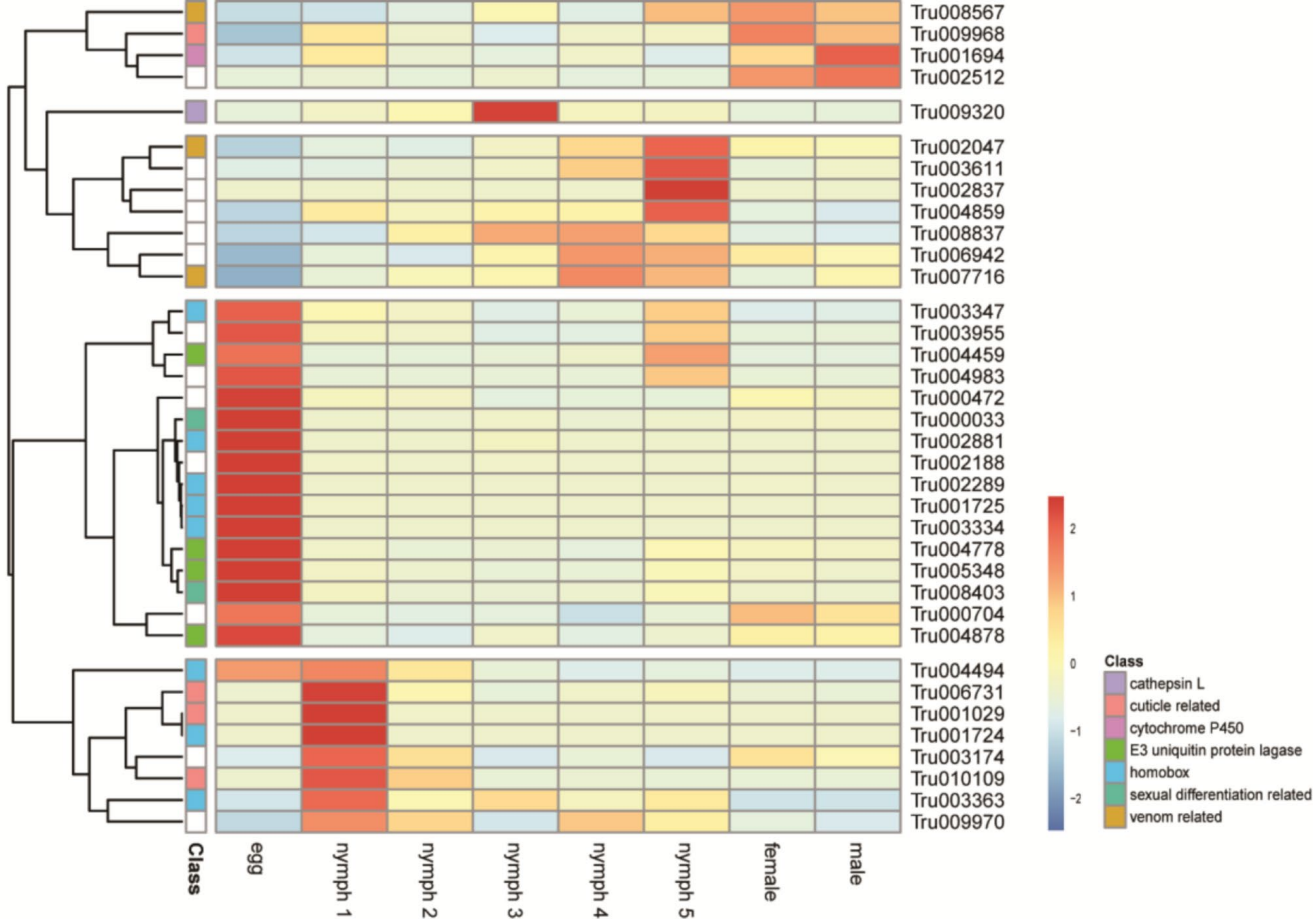
Temporal expression patterns of development-related genes. Red indicates upregulation, and black indicates downregulation. Normalized TPM values were used

Development-related genes, such as *homeobox* protein genes, venom-related genes, and *E3 ubiquitin-protein ligase* genes, play crucial regulatory roles during developmental processes. Expression analysis reveals that these genes exhibit stage-specific expression patterns throughout development (Fig. 7).

In egg stage, *homeobox* genes such as *homeobox protein Nkx-6.2-like* (*Tru004494*), *homeobox protein abdominal-B isoform X1* (*Tru003347*), *homeobox protein Hox-A3-like* (*Tru002881*), *Hox-B4-like* (*Tru003334*), *homeobox protein aristaless-like isoform X1* (*Tru001725*), *homeobox protein SIX6-like* (*Tru002289*), and *retinal homeobox protein Rx1-like isoform X2* (*Tru001724*), along with *E3 ubiquitin-protein ligase* genes (*Tru004778*, *Tru004459*, *Tru004878*, *Tru005348*), and sexual differentiation-related genes, including *male-specific lethal 1-like 1 isoform X3* (*MSL*, *Tru000033*), *transformer-2 protein homolog beta-like isoform X2* (*tra-2*, *Tru008403*), and *doublesex- and mab-3-related transcription factor A2-like* (*dsx*, *Tru010813*), are highly expressed.

At the nymphal stages, specific genes exhibit elevated expression. *Chitinase 3* (*Tru009968*) and cuticle protein-related genes, such as *larval cuticle protein A2B-like* (*Tru001029*, *Tru006731*) and *cuticle protein 19-like* (*Tru010109*), are highly expressed in the first instar nymph (N1). *Cuticle protein 19-like* (*Tru010109*) also maintains high expression in the second instar nymph (N2). In the third instar nymph (N3), *cathepsin L* (*Tru009320*) shows peak expression. The *venom histidine phosphatase-like protein 1* (*Tru007716*) is predominantly expressed in the fourth instar nymph (N4).

During the final developmental transition from nymph to adult, venom-related genes, including *venom histidine phosphatase-like protein 1* (*Tru007716*), *venom serine carboxypeptidase-like* (*Tru002047*), and *venom triabin-like protein 1* (*Tru008567*), as well as *homeobox protein Hox-A1* (*Tru003363*), are highly expressed in the fifth instar nymph (N5).

In adults, gene expression patterns exhibit slight sex-specific differences. *Cytochrome P450 CYP425A1v2* (*Tru001694*) is highly expressed in both sexes, with particularly elevated expression in males.

## Discussion

Beyond its role as a primary vector of Chagas disease in the Americas—where its ability to colonize domestic habitats significantly contributes to local transmission cycles—*T. rubrofasciata* has garnered attention for its pantropical distribution, spanning approximately 45 countries across both the Old and New World [5]. While its epidemiological significance for Chagas disease remains largely restricted to the Americas, its widespread presence in tropical regions raises concerns about potential secondary roles in pathogen transmission. Notably, the complete genome of *T. rubrofasciata* has been sequenced and published [18]. The advent of next-generation sequencing has facilitated genome-wide investigations, enabling the linkage of mRNA sequences to specific biological functions across various tissues, thereby advancing our understanding of key aspects of insect metabolism and physiology [33]. To further elucidate the molecular framework underlying different developmental stages of this species, we performed a comparative transcriptomic analysis.

Principal component analysis (PCA) (Supplementary Fig. 1) distinctly separated the egg stages, nymphal stages, and adult males and females into three groups, highlighting unique transcriptional profiles. In terms of gene expression levels and counts, adult males and females exhibited a slight reduction in the number of expressed genes compared to other stages. In contrast, the egg stage showed a higher average expression level, albeit with fewer highly expressed genes (Fig. 1B, Supplementary Data 2). This pattern aligns with transcriptional dynamics observed in other species during early development. For instance, in *Drosophila melanogaster*, maternal transcripts drive elevated transcriptional activity in eggs, followed by a decline in the number of highly expressed genes as development advances [34]. Similarly, zebrafish embryos exhibit broad activation of developmental regulators at the egg stage, followed by subsequent refinement in later stages [35]. These similarities suggest that the transcriptional activity observed in *T. rubrofasciata* represents a conserved mechanism balancing developmental plasticity and stage-specific functional requirements through specialized gene expression programs critical for early morphogenesis.

Differential expression analysis identified approximately 6,000 differentially expressed genes (DEGs) between the egg stage and nymphal stages, as well as between the egg stage and adults, with the highest number of DEGs occurring between the egg stage and the first two nymphal stages (Fig. 2A; Table 1). This indicates substantial transcriptional changes during the transition from egg to nymph, with these DEGs likely playing key roles across developmental stages. In contrast, the lowest number of DEGs was observed between male and female adults, reflecting similar gene expression patterns in fully developed individuals. Comparisons between the egg stage and all subsequent developmental stages revealed a predominance of upregulated genes, whereas comparisons among the five nymphal stages and the adult stage showed a higher proportion of downregulated genes, indicating that gene expression is more extensive at the egg stage.

Additionally, genes involved in sexual differentiation, such as *tra-2*, *dsx*, and *MSL*, were highly expressed in the egg stage (Fig. 7). *tra-2* regulates the sex-specific splicing of *dsx*, both of which are essential for male and female somatic development within the sexual differentiation pathway [36]. *MSL* genes, critical for dosage compensation in *Drosophila*, are specifically associated with the male X chromosome [37]. These genes have also been reported in the egg stage of other insect species [38, 39]. Our findings provide a temporal framework for transcriptomic analyses that could facilitate the development of male-only lines for pest control applications.

Principal component analysis (PCA) (Supplementary Fig. 1) revealed distinct clustering of egg, nymphal, and adult stages, reflecting their unique transcriptional profiles. Notably, nymph 4 (N4) and nymph 5 (N5) exhibited greater intra-group variation compared to other stages. Several factors may contribute to this biological variability: (1) Developmental Transition: The transition from N4 to N5 represents a critical preparatory phase for adult metamorphosis. In triatomines, late nymphal stages undergo extensive physiological changes, including cuticle remodeling, energy storage, and reproductive organ development, potentially leading to asynchronous gene expression. (2) Feeding Behavior: Previous studies indicate that feeding frequency and duration differ markedly between N4 and N5 in *T. rubrofasciata*. N5 nymphs exhibit prolonged feeding to accumulate nutrients for molting, which may amplify transcriptional heterogeneity. (3) Metabolic Adaptation: The upregulation of lipid metabolism and detoxification-related genes (e.g., cytochrome P450) in N5 (Fig. 7) suggests heightened metabolic activity, possibly influenced by environmental interactions or pathogen exposure. (4) Technical Considerations: Although biological replicates were included in the study, minor batch effects during RNA extraction or sequencing cannot be entirely excluded. However, the consistency of functional enrichment results (e.g., proteasome activity in N5) reinforces the biological relevance of these variations. These findings align with reports in *R. prolixus*, where late nymphal stages exhibit increased transcriptional plasticity to support nutrient acquisition and immune priming [40]. Future studies incorporating larger sample sizes and time-resolved sampling will be valuable in further elucidating the molecular drivers of this variability.

Gene Ontology (GO) functional classification of differentially expressed genes (DEGs) revealed multiple molecular functions, including inorganic cation transmembrane transporter activity, mitochondrial electron transport, and ubiquinol metabolism. Genes associated with catalytic activity and transmembrane transport play essential roles in insect growth and development [41]. KEGG pathway analysis further identified several pathways implicated in developmental processes, categorized as follows: (1) metabolic pathways, such as carbohydrate metabolism and cytochrome P450 metabolism; (2) signaling pathways, including the Notch, p53, and ErbB signaling pathways; and (3) biosynthetic pathways involved in insect hormone biosynthesis, fatty acid biosynthesis, and steroid hormone biosynthesis. Among these, carbohydrate metabolism and cytochrome metabolism are particularly critical for insect growth and development. Carbohydrate metabolism is responsible for energy conversion and storage, whereas cytochrome metabolism facilitates transmembrane transport processes. In *T. rubrofasciata*, signaling pathways play a pivotal role in regulating growth and development. For instance, pathways associated with ecdysteroids (20-hydroxyecdysone, 20E) and juvenile hormones (JH) are key regulators of molting and metamorphosis in insects [42]. Notably, recent studies have highlighted the significance of ecdysteroid signaling in adult insects, particularly in oogenesis, drawing increasing scientific attention [43].

Recent studies indicate that the feeding duration of *T. rubrofasciata* is closely linked to its developmental stage, with first- and second-instar nymphs feeding less frequently than those in the fourth and fifth instars. Notably, the third-instar nymph is the only stage with a feeding duration of less than 15 min [44]. Transcriptomic analysis revealed significant differences in metabolic pathways between stage III nymphs and eggs, particularly in mineral absorption, glycine, serine, and threonine metabolism, as well as the biosynthesis of unsaturated fatty acids. Genes associated with digestion and absorption, including those involved in pancreatic secretion, were upregulated in stage III, along with genes linked to vitamin digestion and absorption, carbon metabolism, and fatty acid degradation. Additionally, *Cathepsin L*, a key enzyme in cellular protein catabolism, exhibited high expression levels at this stage. Previous studies have demonstrated that coleopterans and hemipterans utilize cathepsins for digestion within the gut lumen [45, 46]. However, whether the brief feeding duration observed in stage III is directly related to *Cathepsin L* expression remains to be determined.

The ability of *T. rubrofasciata* to withstand starvation has been previously investigated, revealing a progressive increase in starvation resistance across developmental stages. Stage I juveniles typically survive for approximately 14 to 21 days without food, whereas stage IV juveniles can endure fasting periods ranging from 38 to 120 days [44]. This enhanced starvation resistance may be associated with the downregulation of pathways related to insect hormone biosynthesis, amino acid biosynthesis, and histidine metabolism in fourth-stage nymphs.

Sexual differentiation in insects is governed by a complex interplay of genetic and environmental factors [47]. Genetic determinants include sex chromosome composition, chromosome ploidy, and autosomal elements [48]. In the model insect *D. melanogaster*, sex determination is primarily regulated by *Sex-lethal* (*sxl*), whose alternative splicing is controlled by the exon-splicing activator *Transformer* (*tra*) [37]. *sxl* operates hierarchically with other key sexual differentiation genes, including *tra*, *tra-2*, and *doublesex* (*dsx*) [49]. In *T. rubrofasciata*, *dsx* expression is markedly upregulated in eggs and first-instar larvae, suggesting that sexual differentiation is initiated as early as the embryonic stage. In adults, *dsx* expression exhibits significant sex-specific differences, which correlate with gonadal development and reproductive capacity. The *dsx* proteins function as transcription factors that regulate the expression of cytodifferentiation genes involved in sexual differentiation [50].

In *Drosophila*, *DSXF* and *DSXM* control the expression of *yolk protein* (*Yp*) genes by binding to the fat body enhancer (FBE) [51]. In *Bombyx mori*, ectopic expression of *DSXF1* in males induces female-specific expression of *vitellogenin* (*vg*) and *hexameric storage protein1* (*sp1*), while repressing the male-specific *pheromone-binding protein* (*pbp*) gene. Conversely, ectopic expression of *DSXM1* in females produces the opposite effect [52]. Moreover, *DSXM1* regulates *spitz* (*spi*), an epidermal growth factor receptor ligand, and induces *Abd-B* expression, thereby activating the EGFR signaling pathway essential for male A8 somatic nodal cell proliferation [53]. In *Tribolium castaneum*, *DSXF* promotes *vg* expression, whereas *DSXM* suppresses it [54]. Similarly, in horned beetles, *dsx* is a key regulator of horn development in both dung beetles (*Onthophagus*) and rhinoceros beetles (*Trypoxylus*) [55].

Although the overall structure of *dsx* is evolutionarily conserved, its functional roles and regulatory pathways vary across species. Disruptions in these pathways can lead to severe developmental defects or sex ratio imbalances. Consistently, our findings demonstrate that *dsx* exhibits similar sex-specific expression patterns in *T. rubrofasciata*, reinforcing its pivotal role in sexual differentiation. These insights not only deepen our understanding of sex determination mechanisms in *T. rubrofasciata* but also provide potential targets for novel pest control strategies.

Transcriptomic studies have provided new insights into triatomine research, offering valuable tools for investigating their physiology, immune system, sensory apparatus, taxonomy, and systematics [56]. Latorre-Estivalis et al. (2022) analyzed transcriptomes from the antennae of different *Rhodnius prolixus* developmental stages, demonstrating that neuropeptide gene transcripts, including G protein-coupled receptors (GPCRs) and nuclear receptors, are expressed in the antennae [57, 58]. Similarly, Ons et al. (2016) explored whole transcriptomes of *R. prolixus*,* T. dimidiata*,* T. infestans*, and *T. pallidipennis*, revealing a high degree of sequence conservation in neuropeptide precursors and GPCR genes within the neuroendocrine system of triatomines [59]. Extensive research has been conducted on the transcriptome of triatomine saliva and salivary glands, leading to the identification of key molecules such as palidipine, triabine, procaline (a salivary allergen), and numerous Kazal-type proteins [60–63]. Notably, Mizushima et al. performed a salivary gland transcriptome analysis of the Asiatic *T. rubrofasciata*, identifying abundant homologs of antigen-5, Kazal-type proteins, inositol polyphosphate 5-phosphatase, and apyrase/5’-nucleotidase in its saliva [63].

Transcriptomic analyses of various tissues, including ovarian and testis tissues, digestive tracts, Malpighian tubules, brain, adipose body, and salivary glands, have elucidated genes involved in reproduction and innate immune responses in triatomines. These studies have identified conserved elements such as piwi-interacting RNAs (piRNAs), lysozymes, and key components of the TOLL and Jak-STAT signaling pathways [64, 65]. In our study, we examined *T. rubrofasciata* across different developmental stages and identified *homeobox* protein genes, venom-related genes, and E3 ubiquitin-protein ligase genes as potential developmental regulators. Additionally, *MLS*,* tra-2*, and *dsx* were identified as candidate genes for sexual differentiation. The differentially expressed genes (DEGs) identified in this study could serve as targets for novel control strategies against this blood-sucking pest. The stage-specific expression of *dsx* and *tra-2* in eggs suggests that early intervention strategies, such as RNA interference (RNAi)-coated egg traps, could prevent sexual maturation. Meanwhile, the nymph-specific upregulation of cathepsin L highlights a potential window for targeting feeding behavior. In adults, reliance on *CYP425A1v2* for detoxification supports the development of synergistic insecticides [66]. These strategies, previously validated in model insects, could be adapted for *T. rubrofasciata* with minimal off-target effects.

## Conclusions

In this study, we present the first comprehensive transcriptome analysis covering all seven developmental stages of *Triatoma rubrofasciata*, a key vector of Chagas disease. This work fills a critical gap in triatomine research, as prior studies have largely focused on single tissues (e.g., salivary glands) or limited developmental phases in related species. By integrating stage-specific expression profiles, we identified both conserved and novel regulatory networks governing development and sexual differentiation. Notably, the high expression of *homeobox* protein genes (e.g., *Hox-A3-like*, *Hox-B4-like*) in eggs and nymphs suggests a role in early morphogenesis, a pattern previously observed in *R. prolixus* but not yet characterized in *T. rubrofasciata*. Furthermore, the stage-specific upregulation of *dsx* and *tra-2*, key regulators of insect sex determination, provides new evidence that sexual differentiation may initiate as early as the egg stage in triatomines—diverging from mechanisms described in *Drosophila* and beetles.

Beyond its evolutionary insights, this study has practical implications. The identification of venom-related genes (e.g., *venom histidine phosphatase-like protein 1*) in late nymphal stages correlates with their increasing hematophagous activity, offering potential molecular targets for disrupting feeding behavior. Additionally, the stage-specific expression of *cytochrome P450 CYP425A1v2* in adults highlights metabolic adaptations relevant to pesticide resistance, an emerging challenge in triatomine control. By bridging fundamental genomics with applied entomology, our findings expand the molecular toolkit for triatomine research and provide a foundation for stage-specific interventions aimed at controlling *T. rubrofasciata* populations and mitigating disease transmission.

## Electronic supplementary material

Below is the link to the electronic supplementary material.

Supplementary Material 1

## Data Availability

The transcriptome sequencing data generated in this study are available in the NCBI database under accession number PRJNA1165018 (access link: https://www.ncbi.nlm.nih.gov/bioproject/PRJNA1165018, raw reads are accessible at SRA (https://www.ncbi.nlm.nih.gov/sra?term=PRJNA1165018). Additional datasets and materials supporting the findings of this study are provided within the article and its supplementary files. Further data requests can be directed to the corresponding author (liuqin@nipd.chinacdc.cn).

## Abbreviations

ApoL-1: Apolipoprotein L1
DAS: Differential Alternative Splicing
DEGs: Differentially Expressed Genes
DSXF: Doublesex Female
DSXM: Doublesex Male
FDR: False Discovery Rate
GAPDH: Glyceraldehyde-3-phosphate Dehydrogenase
GO: Gene Ontology
KEGG: Kyoto Encyclopedia of Genes and Genomes
LSD: Least Significant Difference
MSL: Male-Specific Lethal
PCA: Principal Component Analysis
PPI: Protein-Protein Interaction
RT-qPCR: Real-Time Quantitative Polymerase Chain Reaction
STEM: Short Time-series Expression Miner
